# Competition assays and physiological experiments of soil and phyllosphere yeasts identify *Candida subhashii* as a novel antagonist of filamentous fungi

**DOI:** 10.1186/s12866-016-0908-z

**Published:** 2017-01-05

**Authors:** Maja Hilber-Bodmer, Michael Schmid, Christian H. Ahrens, Florian M. Freimoser

**Affiliations:** 1 Agroscope, Institute for Plant Production Sciences IPS, Schloss 1, P.B., 8820 Wädenswil, Switzerland; 2 SIB, Swiss Institute of Bioinformatics, Wädenswil, Switzerland

**Keywords:** Antagonism, Biocontrol, *Candida*, Fungal plant pathogen, Mitochondrial genome, Plant protection, Yeast

## Abstract

**Background:**

While recent advances in next generation sequencing technologies have enabled researchers to readily identify countless microbial species in soil, rhizosphere, and phyllosphere microbiomes, the biological functions of the majority of these species are unknown. Functional studies are therefore urgently needed in order to characterize the plethora of microorganisms that are being identified and to point out species that may be used for biotechnology or plant protection. Here, we used a dual culture assay and growth analyses to characterise yeasts (40 different isolates) and their antagonistic effect on 16 filamentous fungi; comprising plant pathogens, antagonists, and saprophytes.

**Results:**

Overall, this competition screen of 640 pairwise combinations revealed a broad range of outcomes, ranging from small stimulatory effects of some yeasts up to a growth inhibition of more than 80% by individual species. On average, yeasts isolated from soil suppressed filamentous fungi more strongly than phyllosphere yeasts and the antagonistic activity was a species-/isolate-specific property and not dependent on the filamentous fungus a yeast was interacting with. The isolates with the strongest antagonistic activity were *Metschnikowia pulcherrima*, *Hanseniaspora sp.*, *Cyberlindnera sargentensis*, *Aureobasidium pullulans*, *Candida subhashii*, and *Pichia kluyveri*. Among these, the soil yeasts (*C. sargentensis, A. pullulans, C. subhashii*) assimilated and/or oxidized more di-, tri- and tetrasaccharides and organic acids than yeasts from the phyllosphere. Only the two yeasts *C. subhashii* and *M. pulcherrima* were able to grow with N-acetyl-glucosamine as carbon source.

**Conclusions:**

The competition assays and physiological experiments described here identified known antagonists that have been implicated in the biological control of plant pathogenic fungi in the past, but also little characterised species such as *C. subhashii*. Overall, soil yeasts were more antagonistic and metabolically versatile than yeasts from the phyllosphere. Noteworthy was the strong antagonistic activity of the soil yeast *C. subhashii*, which had so far only been described from a clinical sample and not been studied with respect to biocontrol. Based on binary competition assays and growth analyses (e.g., on different carbon sources, growth in root exudates), *C. subhashii* was identified as a competitive and antagonistic soil yeast with potential as a novel biocontrol agent against plant pathogenic fungi.

**Electronic supplementary material:**

The online version of this article (doi:10.1186/s12866-016-0908-z) contains supplementary material, which is available to authorized users.

## Background

The fungal kingdom includes important plant pathogens that cause a plethora of diseases in all crops worldwide. Of particular concern are rot and wilt diseases caused by soilborne fungi, fungal spots, blights and blotches, rusts, mildews, cankers and anthracnoses, as well as postharvest decay of fruits and vegetables [1, 2]. Infestations by aggressive, fungal pathogens can severely constrain agricultural production and often the only resort is crop rotation, fallow, or even an abandonment of the cropland [3, 4].

Soil, roots, and the phyllosphere harbour complex microbiomes consisting of thousands of bacterial and fungal species that may suppress diseases, act as pathogens, or affect plant health and growth by various other mechanisms [5–8]. Yet, microbiomes are still a largely untapped resource for protecting crop plants against pathogens and for increasing agricultural productivity [9, 10]. Considerable efforts are therefore undertaken to harness and use microbiota for novel applications in agriculture [11–14]. Microbiomes of plants, rhizosphere, or soil have been elucidated by large-scale, DNA sequencing-based metagenomics approaches [7, 15, 16], but the contributions and functions of the large majority of the species are still mostly unknown. Microbiota thus consist predominantly of yet uncharacterized bacteria and fungi, tritagonists, that regulate microbial interactions [17].

Yeast-like fungi inhabit all aerobic environments; from the arctic and glaciers to the tropics or even the desert and from dry to saline and high-sugar habitats [18–24]. Many yeast species are particularly well known for their biotechnological applications or medical relevance. In agriculture, yeasts have been identified as powerful antagonists of fungal pathogens causing postharvest and storage diseases and of microorganisms attacking flowers and leaves [25–31]. Few yeast species have reached the market as commercial products for the postharvest control of pathogens (e.g., *Aureobasidium pullulans* as BoniProtect, *Candida oleophila* strain 1-182 as Aspire^TM^, *Candida sake* as Candifruit, *Metschnikowia fructicola* as Shemer, or *Cryptococcus albidus* as YieldPlus) or against fireblight (e.g., *A. pullulans* as BlossomProtect); some of which are not marketed anymore or only registered locally [32–37]. Yeasts suppressing soilborne pathogens have been described rarely and a commercial application has not been considered yet. *Candida valida*, *Rhodotorula glutinis* and *Trichosporon asahii* protected sugar beet against the soil pathogen *Rhizoctonia solani* [38]. In another study, *Saccharomyces unispora* and *Candida steatolytica* antagonised *Fusarium oxysporum* causing wilt disease in kidney beans [39] and *Saccharomyces cerevisiae* controlled a *Fusarium* infection of sugar beet [40]. In a successful example of postharvest biocontrol, *M. fructicola* has been employed as part of a combined strategy to control the soilborne pathogen *Thielaviopsis basicola* on carrots [41]. These examples clearly document the potential of yeasts to suppress and antagonise soilborne pathogens, but also highlight the limited knowledge on their biological functions in soil.

The genus *Candida* comprises several species that have been studied extensively with respect to biotechnological applications, biocontrol, but also as human pathogens. *Candida guilliermondii* is a ubiquitously present, saprophytic yeast that has received particularly broad attention because of its presence in clinical samples, the biotechnological production of metabolites and enzymes, applications in bioremediation, or the control of plant pathogenic fungi [42]. The antagonistic potential of *C. guilliermondii* against diverse fungal pathogens (e.g., *Botrytis cinerea*, *Colletotrichum capsici*, *Penicillium expansum*, *Penicillium digitatum*, *Rhizopus stolonifer*) has been demonstrated in various cultures such as apple, citrus, nectarine, peach, or tomato ([42], and references therein). Other *Candida* species have also been studied for their biocontrol potential and as commercial plant protection agents against postharvest decay of fruits, based on *Candida* species, have been developed (see above) [29, 43, 44].

In the course of the work described here, we used binary competition assays to determine the antagonistic activity of soil and phyllosphere yeasts from Switzerland against a range of pathogenic and saprophytic filamentous fungi. Among the six most antagonistic yeasts out of a collection of 40 different isolates (*A. pullulans, Candida subhashii, Cyberlindnera sargentensis, Hanseniaspora sp., Metschnikowia pulcherrima*, and *Pichia kluyveri*), *C. subhashii* was the only one that has so far not been studied with respect to biocontrol. This species has only been reported from a patient suffering from peritonitis during a long-term peritoneal dialysis treatment and an isolate highly similar to this type strain (99.8% identity in the 26S rDNA D1/D2 domain, 1.3% sequence difference for the 5.8S-ITS region) was obtained from a soil sample from East Japan [45, 46]. Except for these two reports, only one additional publication reporting the mitochondrial genome of *C. subhashii* has appeared [47]. Here, we describe *C. subhashii* as a common and frequent soil fungus that has broad metabolic capabilities, grows in root exudates, and that strongly antagonizes a wide range of filamentous fungi (all species tested in this study, including notorious plant pathogens, saprophytes, but also other antagonists of the genus *Trichoderma*). Since it has not been experimentally confirmed that *C. subhashii* is indeed a pathogen, and based on its broad distribution in different soils and the apparent adaptations to the soil environment, it is concluded that *C. subhashii* is a competitive soil fungus and potential candidate for the biological control of soilborne fungal pathogens.

## Methods

### Isolation and cultivation of fungi

Soil or plant material (e.g., apple leaves, flowers, bark, skin) was diluted 10-fold (*w/v*) with peptone water (1 g/L Bacto Peptone) [48], vigorously mixed, and shaken (20 min, 25 °C, 250 rpm, on an orbital shaker). The resulting suspensions were diluted and different dilutions (usually 1:50 and 1:100) were plated on Difco^TM^ potato dextrose agar (PDA; Becton, Dickinson and Company, Le Pont de Claix, France) supplemented with 5 ml chloramphenicol and tetracycline HCl (5 mg/ml in ethanol or water, respectively), and incubated at 22 °C for 2–4 days. Single fungal colonies were transferred to PDA agar plates without antibiotics and repeatedly streaked out until pure cultures were obtained. Isolates were maintained on PDA agar plates and stored in 15% (*v/v*) glycerol at −80 °C.

### Identification of fungal isolates

Species identification was first attempted by MALDI-TOF as previously described [49]. In cases where MALDI-TOF did not allow species identification, the fungal ITS region was amplified with primers ITS1f [50] and ITS4 [51], PCR products were directly used for sequencing, and all isolates were assigned a species hypothesis according to the UNITE database [52, 53] (see also Table 1). Crude protein extracts of isolates that were identified based on their ITS sequence were used to generate reference MALDI-TOF spectra for future identifications of the same species [49]. All isolates generated in the course of this study have been deposited and are available at the Culture Collection of Switzerland (CCoS; https://www.ccos.ch; Table 1).

**Table 1:** Yeasts and filamentous fungi used in this study. All strains that were isolated in the course of this study are deposited and available at the Culture Collection of Switzerland (CCoS; https://www.ccos.ch)

	Culture Collection	Isolate	SH-number	Name	Source	Origin/Reference
40 yeast isolates tested in this study	CCOS995	BC 1.01	SH216366.07FU	*Rhodosporidium sphaerocarpum*	Agricultural soil	This study, Switzerland
	CCOS996	BC 1.03	SH218818.07FU	*Candida subhashii*	Agricultural soil	This study, Switzerland
	CCOS997	BC 1.06	SH196641.07FU	*Trichosporon dehoogii*	Agricultural soil	This study, Switzerland
	CCOS998	BW 2.02	SH195538.07FU	*Trichosporon ovoides*	Agricultural soil	This study, Switzerland
	CCOS999	BW 5.01	SH196643.07FU	*Trichosporon moniliiforme*	Agricultural soil	This study, Switzerland
	CCOS1000	BW 7.01 A	SH190095.07FU	*Schwanniomyces yamadae*	Agricultural soil	This study, Switzerland
	CCOS1001	BW 7.02	SH182010.07FU	*Trichosporon gracile*	Agricultural soil	This study, Switzerland
	CCOS1009	SHA 10.3	SH175136.07FU	*Candida sp*	Agricultural soil	This study, Switzerland
	CCOS1010	SHA 15.4	SH205045.07FU	*Cryptococcus laurentii*	Agricultural soil	This study, Switzerland
	CCOS1011	SHA 17.2	SH195578.07FU	*Cyberlindnera saturnus*	Agricultural soil	This study, Switzerland
	CCOS1012	SHA 25.3	SH031361.07FU	*Barnettozyma vustinii*	Agricultural soil	This study, Switzerland
	CCOS1013	SHA 43.1	SH216362.07FU	*Rhodotorula graminis*	Agricultural soil	This study, Switzerland
	CCOS1014	SHA 51.1	SH212824.07FU	*Guehomyces pullulans*	Agricultural soil	This study, Switzerland
	CCOS1015	SHA 7.1	SH175136.07FU	*Candida sp*	Agricultural soil	This study, Switzerland
	CCOS1008	NBB 7.2.1	SH195774.07FU	*Aureobasidium pullulans*	Orchard soil	This study, Switzerland
	CCOS1004	FGA 2.2	SH218818.07FU	*Candida subhashii*	Potting soil	This study, Switzerland
	CCOS1005	FGA 3.3	SH196641.07FU	*Trichosporon dehoogii*	Potting soil	This study, Switzerland
	CCOS1006	KS 1/d7.18	SH192275.07FU	*Candida boidinii*	Old compost	This study, Germany
	CCOS1003	F 2.6	SH198057.06FU	*Cryptococcus heimaeyensis*	Irrigation water	This study, Switzerland
	CCOS1002	Dip141103.2	SH199823.07FU	*Pichia membranifaciens*	Insect *(Drosophila)*	This study, Switzerland
	CCOS976	APC 1.1	SH194776.07FU	*Rhodotorula slooffiae*	Apple flowers	This study, Switzerland
	CCOS977	APC 1.10	SH005240.07FU	*Dioszegia sp*	Apple flowers	This study, Switzerland
	CCOS978	APC 1.2	SH180747.07FU	*Metschnikowia pulcherrima*	Apple flowers	This study, Switzerland
	CCOS979	APC 1.5	SH221435.07FU	*Cryptococcus wieringae*	Apple flowers	This study, Switzerland
	CCOS980	APC 1.7	SH192046.07FU	*Basidiomycota sp*	Apple flowers	This study, Switzerland
	CCOS981	APC 10.2	SH207120.07FU	*Basidiomycota sp*	Apple leaves	This study, Switzerland
	CCOS982	APC 11.10 B	SH204094.07FU	*Pichia kluyveri*	Apple bark	This study, Switzerland
	CCOS983	APC 11.3	SH019470.07FU	*Tremella moriformis*	Apple bark	This study, Switzerland
	CCOS984	APC 12.1	SH177122.07FU	*Hanseniaspora sp.*	Apple bark	This study, Switzerland
	CCOS985	APC 13.2	SH194503.07FU	*Sporidiobolales sp*	Apple bark	This study, Switzerland
	CCOS986	APC 18.3	SH194739.07FU	*Erythrobasidium hasegawianum*	Apple leaves	This study, Switzerland
	CCOS987	APC 19.2	SH194775.07FU	*Rhodotorula pinicola*	Apple leaves	This study, Switzerland
	CCOS988	APC 2.3	SH204123.07FU	*Starmerella bombicola*	Apple flowers	This study, Switzerland
	CCOS989	APC 27.4	SH206552.07FU	*Cryptococcus cerealis*	Apple bark	This study, Switzerland
	CCOS990	APC 3.4	SH205935.07FU	*Sporobolomyces oryzicola*	Apple flowers	This study, Switzerland
	CCOS991	APC 6.7	SH193763.07FU	*Leucosporidiella creatinivora*	Apple leaves	This study, Switzerland
	CCOS992	APC 9.2	SH181628.07FU	*Cryptococcus victoriae*	Apple leaves	This study, Switzerland
	CCOS993	AS 1.02	SH181630.07FU	*Cryptococcus sp*	Apple fruit	This study, Germany
	CCOS994	AS 1.06	SH190089.07FU	*Debaryomyces prosopidis*	Apple fruit	This study, Germany
	EUROSCARF	BY4741	-	*Saccharomyces cerevisiae*	-	[59]
16 test strains (filamentous fungi)	CCOS1018	BC 4.14	SH216250.07FU	*Mycosphaerella tassiana*	Agricultural soil	This study, Switzerland
	CCOS1019	BC 8.11	SH207825.07FU	*Trichoderma ghanense*	Agricultural soil	This study, Switzerland
	CCOS1020	BC 8.14	SH213620.07FU	*Gibberella fujikuroi*	Agricultural soil	This study, Switzerland
	CCOS1022	SHA 18.1	SH188374.07FU	*Mucor moelleri*	Agricultural soil	This study, Switzerland
	CCOS1023	SHA 9.1	SH185778.07FU	*Mucor circinelloides*	Agricultural soil	This study, Switzerland
	CCOS1007	NBB 2.4.2	SH190868.07FU	*Trichoderma spirale*	Orchard soil	This study, Switzerland
	CCOS1021	F 2.1	SH181342.07FU	*Trichoderma viride*	Irrigation water	This study, Switzerland
	CCOS1017	Asp 1.1	SH219673.07FU	*Fusarium proliferatum*	Infected asparagus	This study, Switzerland
		A 06.5	SH215493.07FU	*Alternaria eichhorniae*	Diseased, stored apple	Laimburg, Italy
	-	FP13013		*Fusarium poae*	Oat	S. Vogelgsang, Agroscope
	-	FL13014		*Fusarium langsethiae*	Oat	S. Vogelgsang, Agroscope
	CBS 121292	FG0410		*Fusarium graminearum*	Wheat	S. Vogelgsang, Agroscope
	-	FCr11115		*Fusarium crookwellense*	Wheat	S. Vogelgsang, Agroscope
	-	11SD14	-	*Monilinia fructicola*	Infected apricot	[49]
	-	106	-	*Rhizoctonia solani*		[107]
	ARSEF 1095	F52/Met52	-	*Metarhizium brunneum*	*Cydia pomonella*	Austria

### Quantification of yeast antagonism against filamentous fungi in vitro

Yeasts were collected from a PDA plate (less than 2 weeks old), diluted in water, and adjusted to an OD_600_ of 0.1. Fifteen microlitre of this suspension was plated on PDA plates (5.5 cm in diameter) in quadruples. Conidia of filamentous fungi were collected in water, diluted (OD_600_ = 0.1), and 5 μl were inoculated in the centre of the plates (previously overlaid with yeasts or fresh PDA plates as a control). Plates were incubated at 22 °C for 3 to 15 days depending on the fungal species. Growth of the filamentous fungus was quantified before it reached the edge of the control plate (plate without yeasts) with the help of a planimeter (Planix 5, Tamaya Technics Inc., Tokyo, Japan). The average of the relative growth (growth in presence of yeast/growth on control plate) of four replicates for each of the 640 combinations was calculated, log_2_-transformed, and all data were clustered using EPCLUST (http://www.bioinf.ebc.ee/EP/EP/EPCLUST/) for visualisation (correlation measure based distance (uncentered), complete linkage).

### Growth analysis of yeasts at different temperatures

Yeasts were collected from a PDA plate, resuspended in sterile water, adjusted to an OD_600_ of 1, and 10-fold dilutions were prepared in a microtiter plate. The dilutions were spotted onto PDA plates with a multi-blot replicator (delivered volume approx. 3 μl) (V & P Scientific, Inc., San Diego, USA). The plates were incubated at temperatures ranging from 15 to 37 °C and the maximal dilution to which the yeast grew was recorded. Each experiment was performed at least twice for each isolate and the average fold-dilution is indicated as reflective of the growth.

### Microarray phenotype analysis

Overnight liquid cultures were grown in Difco^TM^ potato dextrose broth (PDB; Becton, Dickinson and Company, Le Pont de Claix, France). Cells were pelleted by centrifugation (4 °C, 10 min, 650 g), the supernatant was discarded, and the cells were washed twice with sterile water. For each yeast isolate, a suspension with an OD_600_ of 1 was prepared and 100 μl of this solution were inoculated in each well of a Biolog YT MicroPlate^TM^ (Endotell AG, Allschwil, Switzerland) [54]. The absorption at 590 nm was determined in a plate reader (Infinite® 200 Pro; Tecan Group Ltd., Switzerland) daily for 3 days. All data were normalized with the corresponding water control and growth was expressed relative to the initial measurement at day 0. The maximal relative growth at any of the three time-points was recorded (rounded to the first integer). For each yeast, the experiment was performed twice and the average of the two measurements is shown. Substrates that did not lead to detectable growth for any of the yeasts are not shown. For four carbon sources (glucose, maltose, N-acetyl-glucosamine, melezitose), the microarray phenotype results were confirmed by performing growth analyses in defined medium. Yeast nitrogen base (with amino acids and ammonium sulphate) was supplemented with glucose, maltose, N-acetylglucosamine or melezitose (stock solutions were filter sterilized, final concentration 10 g/L) and growth was followed by measuring the OD_600_ in a plate reader (Infinite® 200 Pro; Tecan Group Ltd., Switzerland). The final measurement (mean of five replicates and standard error) after 42 h is shown.

### Growth in root exudates

Mung bean (*Vigna radiata*) root exudates were collected according to Barbour et al. [55] and used at a final concentration of 0.1 mg/ml. Yeasts were inoculated to an initial OD_600_ of 0.1 and growth was measured in a plate reader (Infinite® 200 Pro; Tecan Group Ltd., Switzerland) for 3 days. The mean of six replicates and the standard error are shown.

### Sequencing and analysis of the C. subhashii FGA 2.2 mitochondrial genome

*Candida subhashii* strain FGA 2.2 genomic DNA was extracted using the Qiagen DNeasy Plant Mini Kit and sequenced on the PacBio RS II platform (performed at the Functional Genomics Center Zurich). Subsequent *de novo* genome assembly and resequencing were performed using PacBio SMRT Portal 2.3.0 [56]. Assembly was generated using protocol RS_HGAP_Assembly.3. The contig corresponding to the mitochondrial genome revealed a linear DNA molecule. Manual curation was performed to extend both telomeres to their full length of 729 bp, resulting in a mitochondrial DNA (mtDNA) assembly of 29,930 bp. One additional resequencing step was performed using SMRT portal protocol RS_Resequencing.1, which resulted in a mean coverage depth of 567-fold. The *C. subhashii* strain FGA 2.2 mitochondrial genome was annotated by reference to the *C. subhashii* type strain CBS10753 [47].

To construct a phylogenetic tree, the mtDNA sequences of 22 diverse yeast species, selected based on previous studies and the availability of complete and annotated mitochondrial genomes [47], were obtained from NCBI (Table 2). The amino acid sequences of the conserved proteins Atp6, Atp8, Atp9, Cob, Cox1, Cox2 and Cox3 were extracted from the downloaded sequences as well as from the *C. subhashii* mtDNA assembly. Multiple sequence alignment (MSA) using MUSCLE 3.8 [57] and trimming of overhanging sequences ensured that the amino acid sequences of all genes and all 22 strains were of similar length. The amino acid sequences of all proteins were concatenated for every strain and a final MSA with MUSCLE was performed. The resulting alignment of 1743 amino acids was used to create a phylogenetic tree by RAxML 8.1 applying the JTT + Γ model [58]. The phylogeny was tested by performing 100 bootstrap replicates.

**Table 2:** Strain designations and accession numbers of the mitochondrial genomes used for calculating the maximum likelihood phylogeny

Species	Strain	Accession	Comment
*Candida albicans*	*L757*	*JQ864233*	*CTG clade*
*Candida metapsilosis*	*MCO448*	*AY962591*	*CTG clade*
*Candida neerlandica*	*NRRL Y-27057*	*EU334437*	*CTG clade*
*Candida parapsilosis*	*CBS 7157 (SR 23)*	*X74411*	*CTG clade*
*Candida sake*	*CBS 159*	*KC993194*	*CTG clade*
*Candida subhashii*	*FR 392/CBS10753*	*GU126492*	*CTG clade*
*Candida subhashii*	*FGA 2.2/CCOS1004*	*KX781248*	*CTG clade*
*Candida tropicalis*	*CBS 94*	*KC993185*	*CTG clade*
*Debaryomyces hansenii*	*CBS767*	*DQ508940*	*CTG clade*
*Meyerozyma guilliermondii*	*CBS 2030*	*KC993176*	*CTG clade*
*Pichia farinosa*	*CBS7064*	*FN356025*	*CTG clade*
*Candida glabrata*	*ATCC 2001*	*AJ511533*	*WGD clade*
*Saccharomyces castellii*	*NRRL Y-12630*	*AF437291*	*WGD clade*
*Saccharomyces cerevisiae*	*S288c*	*KP263414*	*WGD clade*
*Saccharomyces pastorianus*	*Weihenstephan 34/70*	*EU852811*	*WGD clade*
*Saccharomyces servazzii*	*NRRL Y-12661*	*AJ430679*	*WGD clade*
*Barnettozyma californica*	*CBS 252*	*KC993183*	
*Cyberlindnera jadinii*	*CBS 1600*	*KC993189*	
*Kluyveromyces lactis*	*CBS2359*	*AY654900*	
*Kluyveromyces thermotolerans*	*CBS 6340*	*AJ634268*	
*Wickerhamomyces pijperi*	*CBS 2887*	*KC993192*	
*Yarrowia lipolytica*	*W29*	*AJ307410*	*outgroup*

## Results

### The antagonistic activity of naturally occurring yeasts against filamentous fungi in vitro

**Fig. 1:**
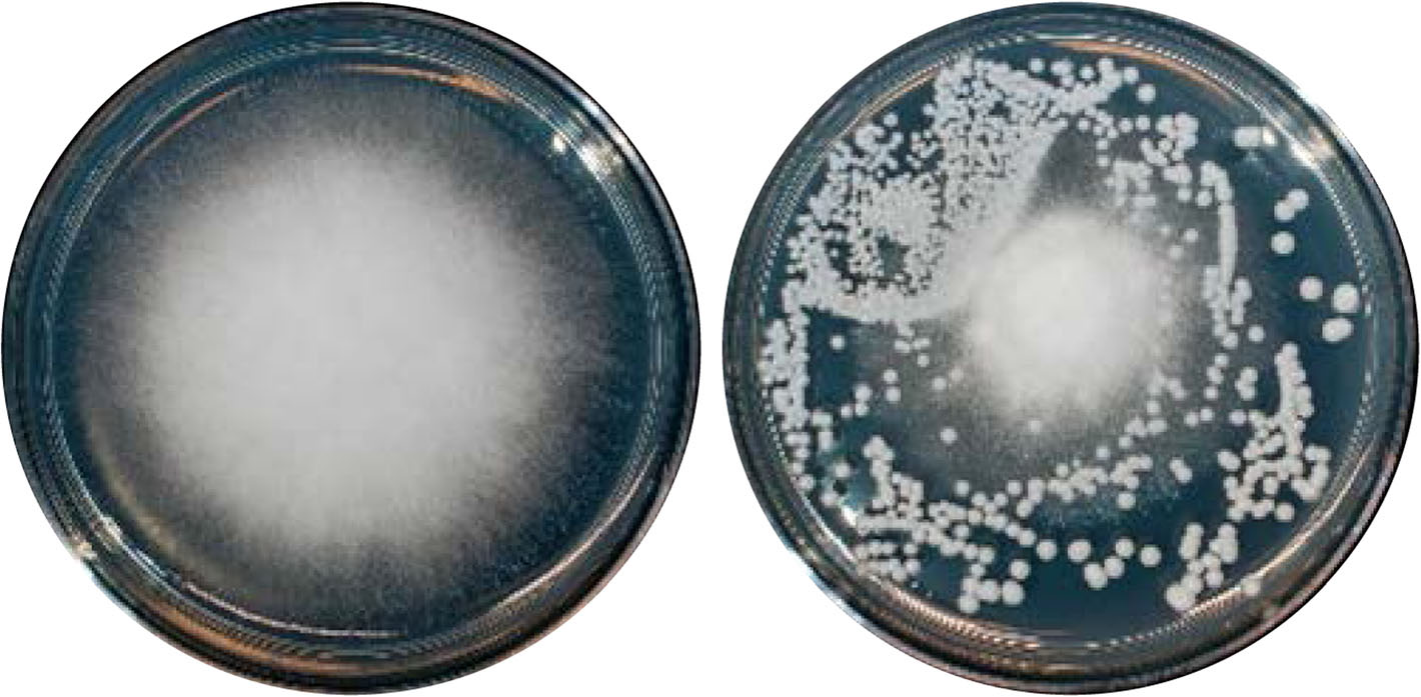
Example of the binary competition assay that was used to quantify the interactions of 40 yeast isolates with 16 filamentous test fungi. Competition assays were performed by quantifying the growth area of a filamentous fungus (e.g., the plant pathogen *Gibberella fujikuroi* BC 8.14) on control plates (*left*) and in the presence of a yeast isolate (e.g., *C. subhashii*, *right*). Overall, 640 competition assays were carried out and quantified (in quadruples)

From a collection of yeasts naturally occurring in agricultural environments, a subset of 40 species was selected (Table 1). These isolates represented the taxonomic diversity in our collection and mostly originated from soil samples (agricultural soil, orchard soil, potting soil, compost; 18 isolates), and the apple phyllosphere (flowers, leaves, fruits, bark; 19 isolates) (Table 1). In addition, one isolate each from irrigation water or a *Drosophila* species (collected in Wädenswil, Switzerland) was included. Finally, for comparison, a reference strain of *Saccharomyces cerevisiae* (BY4741) [59] was included. The antagonistic activity of these 40 yeasts against 16 fungal test strains (a broad selection of commonly isolated, pathogenic, antagonistic, or saprophytic filamentous fungi) (Table 1) was quantified by determining the relative growth of each filamentous fungus in the presence of each yeast (relative to the growth in the absence of yeasts) (Fig. 1; Additional file 1).

**Fig. 2:**
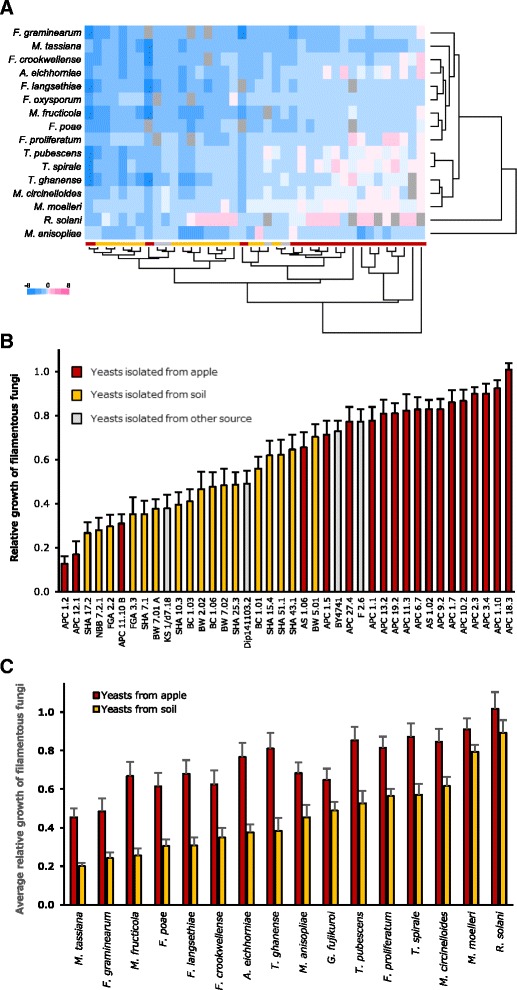
Binary competition assays identify strongly antagonistic yeasts with potential for biocontrol applications. **a** The average relative growth (four replicates) of 16 filamentous fungi in the presence of 40 different yeasts was log_2_-transformed and all data were clustered (correlation measure based distance (uncentered), complete linkage). Colours (see legend) range from strong inhibition (−8; *dark blue*), via no effect (*white*) to strong growth promotion (8; *dark pink*). Missing data are indicated by *grey squares*. **b** The overall average relative growth of filamentous fungi (over all 16 test strains used in this study) in the presence of each yeast isolate. The strain *S. cerevisiae* BY4741 is included as a reference. **c** The average relative growth of each filamentous fungus (average of relative growth in presence all apple or soil yeast isolates). Data obtained with yeasts that were isolated from the apple phyllosphere or from soil are marked in *red* and *yellow*, respectively

All data were clustered based on the outcome of the pairwise interactions of all filamentous fungi with each yeast isolate (Fig. 2a). Overall, the majority of yeast isolates reduced the growth of filamentous fungi, but in a few interactions a small stimulatory effect of a yeast isolate was detected (Fig. 2a; Additional file 1). Based on their growth profiles in the presence of all 40 yeast isolates, the three *Trichoderma* isolates were clustered together with the two *Mucor* isolates, while nine plant pathogenic species (six *Fusarium* isolates, *Alternaria eichhorniae*, *Mycosphaerella tassiana*, *Monilinia fructicola*) formed a second, broad cluster (Fig. 2a). The growth profiles of *R. solani* and *Metarhizium brunneum* in the presence of yeasts strongly differed from each other and from all other filamentous fungi. Clustering of the different yeasts based on their effect on the growth of all 16 filamentous fungi lead to a clear separation of isolates obtained from the apple phyllosphere and those isolated from soil (Fig. 2a).

The overall average relative growth of filamentous fungi (over all 16 isolates used in this study) in the presence of each yeast isolate revealed a broad spectrum of responses (Fig. 2b). While, on average, some yeast isolates (e.g., APC 18.3) exhibited no detectable effect on filamentous fungi, others (e.g., APC 1.2) reduced their growth by more than 80%. The variance of this measure, for each yeast, was small and similar over the entire range of overall relative growth, suggesting that the average antagonistic activity, against a broad range of filamentous fungi, is an inherent property of a particular yeast isolate. The same effect was documented by ranking all 40 yeasts according to their effect on the relative growth of all 16 filamentous fungi (most antagonistic yeast ranked as “1”; least antagonistic isolated as “40”) (not shown). Based on both measures, the overall average relative growth and the average rank for all filamentous fungi, the same six yeast isolates were identified as having the highest antagonistic activity (APC 1.2: *Metschnikowia pulcherrima*, APC 12.1: *Hanseniaspora sp.,* SHA 17.2: *Cyberlindnera sargentensis*, NBB 7.2.1: *A. pullulans*, FGA 2.2: *C. subhashii*, APC 10.11 B: *Pichia kluyveri*). In 10 interactions with filamentous fungi, *M. pulcherrima* (APC 1.2) was the most antagonistic yeast isolate among those tested here (average relative growth of 0.1, average rank of 1.9). Although the two most antagonistic yeast isolates were obtained from apple (APC 1.2 and APC 12.1), overall the results indicated weaker antagonism of yeasts isolated from apple as compared to the isolates obtained from soil samples (Fig. 2b). Comparing the average relative growth of each filamentous fungus in the presence of yeasts from soil (17 isolates) or from apple (19 isolates) documented this finding: as compared to the apple yeasts, soil yeasts more strongly reduced the growth of all tested filamentous fungi (Fig. 2c). The overall relative growth of the 16 filamentous fungi ranged from 0.3 to 0.9 (average 0.6) and above-ground plant pathogens (e.g., *M. tassiana*, *F. gramineaurm*, *F. poae*, *M. fructicola*, *F. langsethiae*, *F. crookwellense*, *A. eichhorniae*) were generally more sensitive to inhibition by yeasts than soil fungi (Fig. 2c). Fast-growing, saprophytic soil fungi such as *Mucor circinelloides*, *Mucor moelleri*, and the soil pathogen *R. solani* were least inhibited in their growth by yeasts.

### Physiological characteristics of strongly antagonistic yeasts from soil or apple

The six overall strongest antagonists comprised three yeasts from apple (APC 1.2: *M. pulcherrima*; APC 12.1: *Hanseniaspora sp.*; APC 10.11 B: *P. kluyveri*) and soil each (SHA 17.2: *C. sargentensis*; NBB 7.2.1: *A. pullulans*; FGA 2.2: *C. subhashii*). In order to identify common and distinguishing characteristics that may affect the potential as biocontrol agents, these six most antagonistic yeasts were further characterized with respect to their growth requirements.

**Fig. 3:**
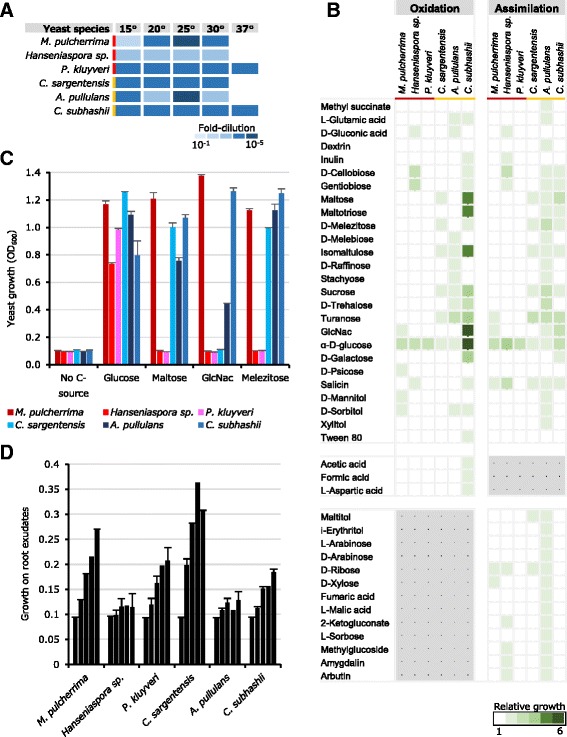
Physiological characteristics of strongly antagonistic yeasts. The six most strongly antagonistic yeasts were characterized by determining their growth at different temperatures (**a**), the assimilation and oxidation of different carbon sources (**b**) and the growth with selected sugars (**c**) or with root exudates (growth at days 0, 1, 2, 3, and 6 is depicted) (**d**). All experiments were repeated at least twice and the mean and standard errors are shown

All six yeast isolates grew well at temperatures up to 30 °C and two isolates, one isolate each from apple and soil (APC 11.10 B: *P. kluyveri*; FGA 2.2: *C. subhashii*, respectively), were able to multiply at 37 °C (Fig. 3a). Microarray phenotype analysis, using the Biolog YT MicroPlate^TM^, revealed a broader metabolic versatility of the three soil yeasts as compared to the three isolates obtained from the apple phyllosphere (Fig. 3b). Most noteworthy were a number of di-, tri- and tetrasaccharides (e.g., maltose, melebiose, palatinose, sucrose, maltotriose, melezitose, raffinose, stachyose) that were assimilated and/or oxidized by at least one soil yeast, while none of these carbon sources were utilized by any of the three yeast isolates obtained from the apple phyllosphere. In particular the two yeasts *A. pullulans* (NBB 7.2.1) and *C. subhashii* (FGA 2.2) assimilated and/or oxidized a large number of compounds (34 and 20, respectively), including different acids (e.g., acetic, formic, aspartic, fumaric, malic acids) (Fig. 3b). In contrast, *P. kluyveri* (APC 11.10 B) only grew with glucose and *M. pulcherrima* (APC 1.2) and *Hanseniaspora sp.* (APC 12.1) only showed detectable growth with 9 and 11 carbon sources, respectively. Interestingly, however, the phyllosphere yeast *M. pulcherrima*, as well as *C. subhashii* and *A. pullulans*, were able to utilize N-acetyl-glucosamine (GlcNac), a component of bacterial and fungal cell walls and insect exoskeletons. The broad metabolic versatility observed, for example for *A. pullulans*, did not go along with the ability to grow with root exudates as the sole source of nutrients (Fig. 3c). *Aureobasidium pullulans* (NBB 7.2.1) and *Hanseniaspora sp.* (APC 12.1) did not grow solely in root exudates (0.1%). In contrast, *P. kluyveri* (APC 11.10 B), which grew only in the presence of glucose in the phenotype microarray analysis, was able to multiply in 0.1% (*w/w*) mung bean root exudate. Of the six yeast isolates tested here, the soil isolate SHA 17.2 of *C. sargentensis* grew best in root exudates.

### *Candida subhashii* is an abundant soil fungus

One of the overall strongest antagonists was *C. subhashii*, a species that has previously only been described in a patient sample in Canada and was considered a human pathogen [45]. During our collection of fungal isolates from Swiss agricultural samples, *C. subhashii* was repeatedly isolated from agricultural soil and from commercially available potting substrates. In one of the latter, *C. subhashii* constituted approx. 50,000 CFU per gram of soil and was the fourth most frequent taxon based on ITS barcode sequencing (data not shown). To further confirm that the *C. subhashii* soil isolate was indeed the same species as the clinical isolate, the mitochondrial genome of the Swiss *C. subhashii* isolate was sequenced (available at NCBI under the accession number KX781248) and phylogenetic analyses were performed.

**Fig. 4:**
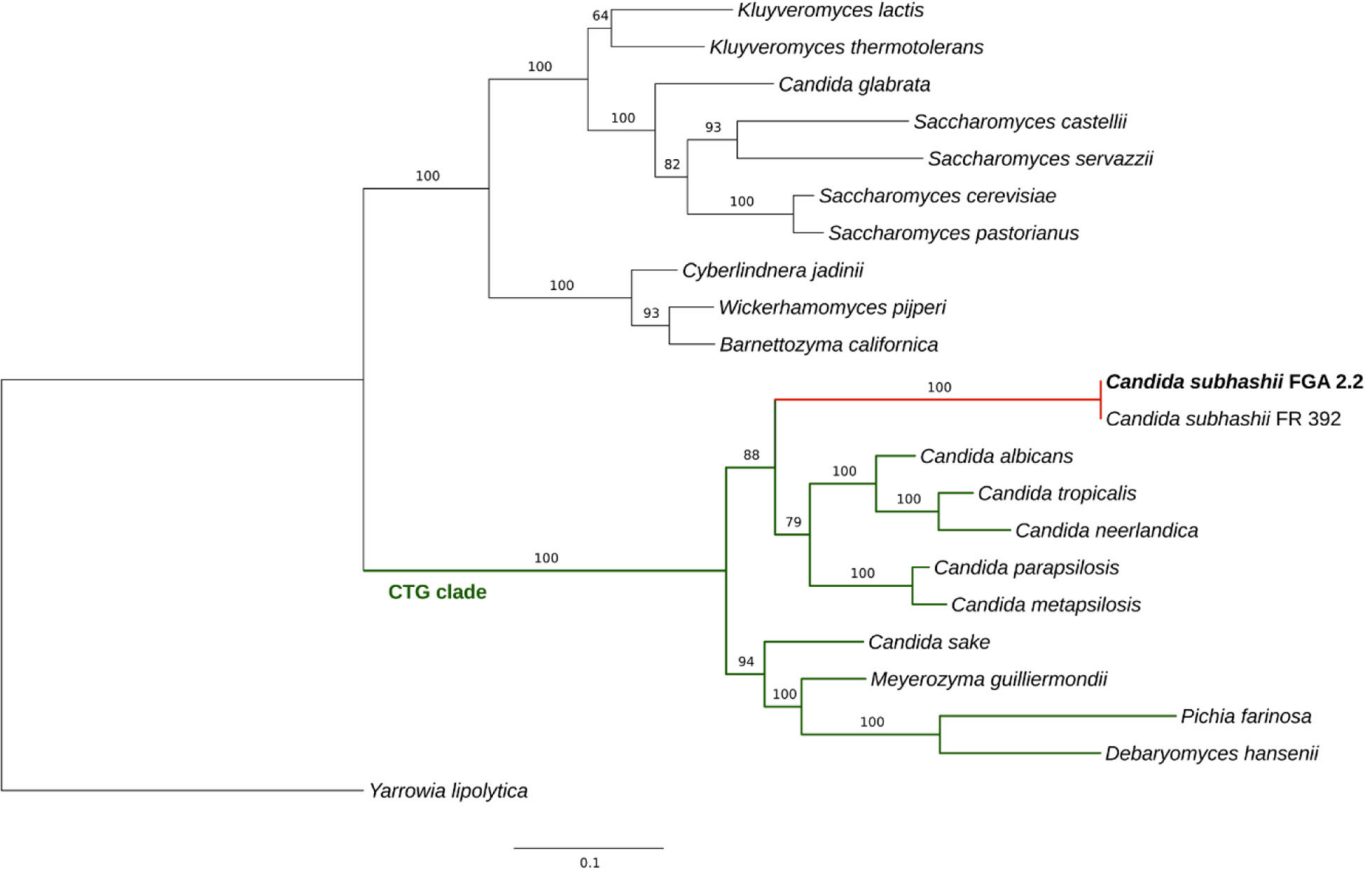
Maximum likelihood phylogeny constructed using a concatenated alignment of conserved mitochondrial protein sequences. The concatenated amino acid alignment of the conserved protein coding genes Atp6, Atp8, Atp9, Cob, Cox1, Cox2 and Cox3 was used to construct a phylogenetic tree. The isolate *C. subhashii* FGA 2.2 is shown in bold and the CTG clade is marked in green. *Yarrowia lipolytica* was used as the outgroup. As the concatenated protein sequences encoded by the seven genes of the previously published and the Swiss *C. subhashii* strains were identical, the branch length on the respective node was zero (in *red*). Bootstrap scores for all nodes are shown (percentage of 100 bootstrap runs). The bar represents the number of amino acid substitutions per site. Further information about the selected sequences is reported in Table 2

The mitochondrial genome sequence of the *C. subhashii* soil isolate FGA 2.2 was identical to the *C. subhashii* type strain (FR 392/CBS 10753), except that the former had an insertion of 135 bp in a non-coding region between two genes (bases 15,872 to 16,006 in the assembly of FGA 2.2). Consequently, the FGA 2.2 mitochondrial genome exhibited the same peculiarities as the corresponding genome of the type strain: exceptionally high GC content (52.7%), a lack of introns in coding sequences, and telomere-like termini of the linear molecules. A maximum likelihood phylogenetic tree of seven mitochondrial proteins (Atp6, Atp8, Atp9, Cob, Cox1, Cox2, Cox3) revealed the *C. subhashii* sequences as a group basal to the *C. parapsilosis/C. albicans/C. tropicalis* cluster, within the CTG clade. The CTG clade comprises the majority of *Candida* species and forms a monophyletic group of yeasts that exhibit a genetic code transition, causing the codon CTG to be translated as serine instead of leucine [60–62] (Fig. 4). Based on these results it was concluded that the two *C. subhashii* isolates indeed belong to the same species, are virtually identical despite the vastly different sources of origin, and that soil is a natural habitat of *C. subhashii*.

## Discussion

### Soil yeasts are generally more antagonistic and metabolically versatile than apple phyllosphere yeasts

Our competition experiments indicated that, on average and under the in vitro conditions tested here, yeasts isolated from soil suppress filamentous fungi more strongly than phyllosphere yeasts. This was the case irrespective of whether the filamentous fungus was isolated from soil or the phyllosphere, or if it was a pathogen or saprophyte. Furthermore, the comparison of three strongly antagonistic yeasts from soil and from the apple phyllosphere suggested a higher metabolic diversity of soil yeasts.

Due to rapidly fluctuating temperatures, low humidity, scarce nutrient availability, and UV irradiation, the phyllosphere is considered a harsh environment [63], but likely features a lower niche complexity as compared to soil. Consequently, interspecific competition between phyllosphere microorganisms is strong and favours the evolution of antagonistic activities to ward off competing microbes. Soil, in contrast, is a highly heterogeneous and rich habitat with a plethora of niches and thus hosts a complex microbiome [64]. In addition to environmental factors and interspecific competition, plants release root exudates and thereby also shape the microbial community in the rhizosphere [6, 65, 66]. The ability to metabolize root exudates may thus indicate adaptation of the corresponding yeast to soil. Indeed, soil yeasts were able to grow in the presence of various sugars and organic acids (e.g., maltose, sucrose, raffinose, acetic, formic, aspartic, fumaric, malic acids) that have been detected in root exudates of higher plants [67], while the tested, strongly antagonistic phyllosphere yeasts were unable to utilize these substrates. Nevertheless, the two phyllosphere yeasts *M. pulcherrima* and *P. kluyveri* were both able to multiply in root exudates, suggesting that on one hand plants likely release factors that allow these species to grow and that on the other hand *M. pulcherrima* and *P. kluyveri* may have the potential to colonize the rhizosphere, even though they were usually isolated from the phyllosphere. This finding is particularly relevant with respect to potential biocontrol applications against soilborne fungal pathogens, where rhizosphere competence is a factor that can contribute to a successful control [68–70].

### Binary competition assays identify strongly antagonistic yeasts with potential for biocontrol

The dual culture assays employed here revealed the antagonistic activity of 40 yeast isolates against 16 filamentous fungi. The level of inhibition ranged from no effect at all (even slight stimulatory activities were detected in some interactions) to a growth reduction of more than 80% as compared to growth on the control plates (in the absence of yeasts). The most strongly inhibitory yeasts were *M. pulcherrima*, *Hanseniaspora sp.*, *C. sargentensis*, *A. pullulans*, *C. subhashii*, and *P. kluyveri*. Except for *C. subhashii*, these species, or close relatives thereof, are known antagonists and have been implicated in the biological control of plant pathogenic fungi in the past. The general nature of the antagonistic activity observed under the experimental conditions used here suggests that yeasts inhibited filamentous fungi based on their strong competitiveness for micro- and/or macro-nutrients or due to indirect effects, which is an advantageous property for a potential biocontrol agent. Further studies will have to reveal the mode of antagonism and to decipher the contribution of competition, indirect effects of metabolites, or specific antagonistic factors, in each interaction, in more detail.

In the experiments described here, *M. pulcherrima* was the overall most strongly antagonistic yeast. Pulcherrimin, an iron-binding pigment produced by *M. pulcherrima*, is believed to mediate this antagonistic activity against other fungi [71–74]. In the past, *M. pulcherrima* has been studied as an antagonist of fruit rot diseases (for example caused by *Alternaria alternata*, *B. cinerea*, *P. expansum*) [31, 75, 76] and a related species, *M. fructicola*, is being used for postharvest biocontrol applications against storage diseases of sweet potatoes and carrots [77]. A strong antagonistic activity against soilborne fungal pathogens and species of *Fusarium* has not been reported. *Hanseniaspora* species are widespread and frequent in the environment, mostly studied with respect to their occurrence on grapes and winemaking, and their antagonistic activity against green mould of citrus or *B. cinerea* was shown [78–82]. *Cyberlindnera sargentensis* (synonym *Williopsis sargentensis*) belongs to a genus of yeasts that have been shown to promote plant growth, produce volatile sulphur compounds, and kill other fungi or bacteria via killer proteins [83–88]. The basidiomycetous yeast *A. pullulans* is a cosmopolitan species that is used in biotechnology and acts as an antagonist against fungal and bacterial plant pathogens such as postharvest diseases or fire blight [25, 89–95]. *Pichia kluyveri* and related species (e.g., *Wickerhamomyces anomalus*, *P. fermentans*, etc.) are widely studied with respect to wine fermentation as well as biological control, mostly of fungal postharvest diseases of fruits [30, 96–101].

Besides the identification of known antagonists (as well as at least one new antagonist; *C. subhashii*), this study also identified soilborne pathogens and several *Fusarium* species as new, potential targets of antagonistic yeasts. The results presented also suggest that yeast antagonism is an isolate-/species-specific property and little dependent on the target organism: a strongly antagonistic yeast exhibits this activity against a broad range of fungi. This finding has important implications for using and studying such yeasts with respect to their application in biocontrol. For example, it may be more promising to optimize the activity of demonstrably strong antagonists than isolating “new” antagonists for each pathogenic fungus to be controlled. An intriguing possibility for optimizing the activity of biocontrol organisms are communities of compatible strains that may achieve better control of plant pathogens than single strains. Initial experiments with mixtures of weakly antagonistic yeasts show that such synergistic effects can indeed be observed (data not shown). With respect to research, these results emphasize the need to study and reveal modes of antagonism that will enable translating strong antagonistic activity in the laboratory to an effective and reliable control in the field. Reliable, biological assays, but also 3rd generation DNA sequencing technologies and bioinformatics tools that have become available as of late, are the foundations for characterizing potential biocontrol strains and for identifying modes of antagonism goal-oriented and rapidly.

### *Candida subhashii* is an antagonistic soil fungus

Among the strongly antagonistic yeasts, *C. subhashii* was the least studied species and not described as an antagonist of saprophytic and pathogenic, filamentous fungi. In fact, *C. subhashii* was considered a human pathogen because it has been isolated from a patient sample [45]. Nevertheless, it must be noted that only one case report of a *C. subhashii* infection exists: a patient on a long-term peritoneal dialysis treatment developed a peritonitis that was ascribed to a *C. subhashii* infection and successfully treated with fluconazole, ampicillin, and amoxicillin [45]. Whether or not *C. subhashii* can indeed colonise and cause symptoms in a healthy mammalian host has not been tested. The thermotolerance (growth at 37 °C) of *C. subhashii*, also observed for the isolates described here and for an isolate similar to *C. subhashii* described from Japan [46], is a requirement for human pathogenicity, but many isolates exhibiting this property, more frequently found within the Ascomycota than the Basidiomycota, have not yet been described as mammalian, let alone human pathogens [102].

Here, *C. subhashii* was repeatedly isolated from soil samples and from commercially available potting substrates, where it occurred in large concentrations (among the most frequent fungi in potting substrate: approx. 50,000 CFU per gram of substrate, the fourth most frequent taxon based on ITS barcode sequencing (data not shown)). In addition, *C. subhashii* was highly competitive against different soil fungi, metabolized carbohydrates commonly found in the rhizosphere, and grew in root exudates as well as on roots and in soil. The metabolic profile of the Swiss *C. subhashii* isolate FGA 2.2 was comparable to the one of *Candida sp.* NY7122, a pentose-fermenting soil yeast that is similar to *C. subhashii* and that was isolated from a Japanese soil. However, the latter isolate was able to assimilate L-arabinose and D-xylose [46], which was not the case for *C. subhashii* FGA 2.2 under the conditions tested here. Based on these results it was concluded that soil is the natural habitat for *C. subhashii*, where this species is a common and competitive organism. Specifically, the particular large number of *C. subhashii* cells in potting substrate, comprised of white and black peat (of European origin), suggests that either or both of these components are a natural reservoir of this antagonistic soil yeast.

The extremely high similarity of the mitochondrial genomes of the Swiss and clinical (Canadian) *C. subhashii* isolates is surprising and unexpected, particularly when considering the vastly different origins of the two isolates. However, identical or almost identical mitochondrial genomes have also been reported, for example, in *Penicillium* isolates from Spain and China, respectively, and may indicate a rapid, global spread of one particular isolate of *C. subhashii* [103]. On the other hand, the identical mitochondrial genomes are in contrast to studies reporting considerable intra-species variation in size, intron content, and recombination in fungal mitochondrial genomes [104–106]. At present, it is not clear why the *C. subhashii* mitochondrial genome is so conserved and future studies will have to address the conservation and evolution of the mitochondrial genome in more detail as well as reveal the entire genome sequence of *C subhashii* as a basis for identifying genes mediating antagonistic functions.

## Conclusions

The work presented here combines a broad screening of the antagonistic activity of naturally occurring yeasts against saprophytic and pathogenic filamentous fungi with growth analyses to compare the metabolic potential of the most antagonistic yeasts. Among the most strongly antagonistic yeasts were *M. pulcherrima*, *A. pullulans*, *Hanseniaspora sp.*, *C. sargentensis*, *P. kluyveri* and *C. subhashii*. Competition assays indicated that the antagonistic activity of yeasts is an inherent property of particular yeast isolates and species and little dependent on the interacting filamentous fungus. Among the strongly antagonistic yeasts, soil yeasts were generally more antagonistic and metabolically versatile as compared to yeasts isolated from the phyllosphere. The identification of *C. subhashii* as a strongly antagonistic soil yeast is particularly noteworthy, because previously the natural habitat of this species was unknown and it was described, in one publication, as a human pathogen. The results presented here thus define *C. subhashii* as a common and competitive soil yeast.

## Additional file

Additional file 1: The average relative growth (four replicates) of all binary competition experiments (16 filamentous fungi in the presence of 40 different yeasts) performed in the course of this study. (XLSX 21 kb)
